# Investigating the effect of plasma activated water on entomopathogenic nematodes under laboratory conditions

**DOI:** 10.1016/j.heliyon.2025.e42038

**Published:** 2025-01-16

**Authors:** Pratik Doshi, Matej Klas, Stanislav Kyzek, Anna Zahoranová, Božena Šerá

**Affiliations:** a Department of Environmental Ecology and Landscape Management, Faculty of Natural Sciences, Comenius University Bratislava, Ilkovičova 6, 84215, Bratislava, Slovak Republic; b Department of Experimental Physics, Faculty of Mathematics, Physics and Informatics, Comenius University Bratislava, Mlynská Dolina, 84248, Bratislava, Slovak Republic; c Department of Genetics, Faculty of Natural Sciences, Comenius University Bratislava, Ilkovičova 6, 84215, Bratislava, Slovak Republic

**Keywords:** Plasma activated water, Entomopathogenic nematodes, *Steinernema*, *Heterorhabditis*, Mortality

## Abstract

Entomopathogenic nematodes are currently being tested for their efficiency in controlling several insect pests. In recent years, non-thermal plasma has been investigated as a state-of-the-art technology for its disinfection/decontamination properties on the seed surface. In addition, it is also used to induce seed germination. In this investigation, the effect of plasma activated water (PAW) was tested on three EPN species, namely *Steinernema feltiae* Filipjev (1934), *S. carpocapsae* Weiser (1955), and *Heterorhabditis bacteriophora* Poinar (1976). Seven different PAW prepared at different treatment times, that is, (1s, 3s, 5s, 10s, 20s, 60s, 90s) were tested directly on the three selected nematode species. Distilled water was used as a control treatment (0s). In the case of *H. bacteriophora*, significantly higher mortality was observed in PAW preparation times of 5, 10, 20, 60 and 90s compared to the control. In the case of *S. feltiae*, significantly high mortality was observed for PAW preparation times of 10, 20, 60 and 90s. However, *S. carpocapsae* was found to have the least sensitivity against all PAW treatments, with a maximum mortality of 14 % (<20 %), indicating the potential synergy between PAW and EPNs. The possibility of combined treatments in the context of integrated pest management is presented and discussed.

## Highlights

• *H. bacteriophora* was least sensitive with significantl high mortality observed in PAW prepared at 5, 10, 20, 60 and 90s.
• In *S. feltiae*, significantly high mortality was observed for PAW preparation times of 10, 20, 60 and 90s.
• *S.carpocapsae* was found to have the least sensitivity against all PAW treatments, with a maximum mortality of 14 % (<20 %).

## 1 Introduction

Agriculture is one of the most important sectors in the world. The use of chemical pesticides is the most conventional method of controlling insect pests and pathogenic plant diseases. With the advancement of research in the field of agriculture and the realization of the side effects of chemical pesticides in the food chain, the search for alternatives to the use of chemical pesticides has become a hotspot for researchers [1,2]. Biological control is perceived as one of the successful alternatives to the use of chemical pesticides. Specifically, entomopathogenic nematodes (EPNs) have been researched and have been quite successful in controlling insect pests in agricultural crops. EPNs are safe for nontarget organisms and were found to be nontoxic to humans [3]. The free-living infective stage is the only one that exists outside the host [4].

EPNs were identified, researched, and commercialized in the 19th century [5]. Two genera of nematodes, *Steinernema* and *Heterorhabditis* (Rhabditida: Steinernematidae and Heterorhabditidae, respectively) belong to the group of EPNs [6]. *Steinernema* spp. and *Heterorhabditis* spp. inhabit naturally in soil and are obligate insect parasites [7]. Due to their association with a wide range of mutualistic bacteria, they are potent killers of insect pests [[8], [9], [10]]. In addition to killing insect pests, the presence of EPNs has been found to induce systemic resistance in plants against plant parasitic nematodes [11]. There are currently more than 100 nematode species belonging to the genus *Steinernema* and 21 nematode species belonging to the genus *Heterorhabditis* [12]. *Heterorhabditis bacteriophora* Poinar, 1975 is the most widely found species with a wide geographical distribution [13]. Several studies have been carried out to show the infectivity of *H. bacteriophora* in different insect pest species such as *Popillia japonica* Newman (Coleoptera: Scarabaeidae) ([14]; Torrini et al., 2020), *Diabrotica virgifera virgifera* LeConte (Coleoptera: Chrysomelidae) [15,16], *Anomala (=Exomala) orientalis* Waterhouse (Coleoptera: Scarabaeidae), *P. japonica* and *Cyclocephala borealis* Arrow (Coleoptera: Scarabaeidae) [17], *Oulema melanopus* Linnaeus (Coleoptera: Chrysomelidae) [18]. *Steinernema carpocapsae* Weiser, 1955 are found to infect *Anoplophora glabripennis* Motschulsky (Coleoptera: Cerambycidae) [19], *Arbela dea* C. Swinhoe (Lepidoptera: Cossidae) [20], *Carposina nipponensis* Walsingham (Lepidoptera: Carposinidae) [21], *Mamestra brassicae* L. (Lepidoptera: Noctuidae) [22], whereas *Steinernema feltiae* Filipjev, 1934 is known for controlling *Chilo infuscatellus* Snellen (Lepidoptera: Crambidae) [23], and *Phyllotreta striolata* Fabricius (Coleoptera: Chrysomelidae) [24].

Plasma is the fourth state of matter consisting of high-energy particles such as electrons, ions, excited atoms, excited molecules, ultraviolet (UV) radiation, free radicals, etc. [25,26]. Due to the inability of nonthermal plasma (NTP) to treat the irregular seed surface evenly, scientists proposed that water exposed to different forms of NTP discharge would generate short- and long-lived reactive species in water, i.e. plasma activated water (PAW) [26] that can be used for seed surface disinfection [27]. PAW contains different reactive particles such as HNO_2_, HNO_3_, and H_2_O_2_ that were studied by Oehmigen et al. [28]. These particles are reactive oxygen species (ROS) comprising (O_3_, ^1^O_2_, O, OH, HO_2_, H_2_O_2_) and reactive nitrogen species (RNS) comprising nitrogen-oxoacid-based RNS (NO, NO_2_^−^, NO_3_^−^, N_2_O_3_, N_2_O_4_, N_2_O_5_) [29]; collectively referred to as reactive oxygen-nitrogen species (RONS) [30].

PAW has found several uses in crop protection in recent years. PAW has been tested against a variety of fungal plant pathogens, for example, in *Fusarium graminearum* [[31], [32], [33]], *Aspergillus* spp. [[34], [35], [36]]; bacterial plant pathogens such as *Xanthomonas vesicatoria* in tomato [37], *Staphylococcus aureus* in kiwifruit [38], *S. aureus* in strawberries [39], *Escherichia coli* on the surface of soyabean seeds [40]. PAW has also been documented to have an insecticidal effect on insect pests such as *Planococcus citri* Risso (Hemiptera: Pseudococcidae) (mealybug) *in vitro* [41], *Tetranychus urticae* Koch (Trombidiformes: Tetranychidae) [42]. Donohue et al. [43] found a positive correlation between atmospheric pressure plasma discharge (APPD) and mortality of western flower thrips (*Frankliniella occidentalis* Pergande (Thysanoptera: Thripidae); tobacco thrips (*Frankliniella fusca* Hinds (Thysanoptera: Thripidae); Asian tiger mosquito, *Aedes albopictus* Skuse (Diptera: Culicidae); two spotted spider mite (*T. urticae*); and German cockroach (*Blattella germanica* Linnaeus (Blattodea: Ectobiidae)).

Few studies have tested the effect of NTP on nematode species. For example, Mitsugi et al. [44] tested the surface barrier discharge ozone generator against *Caenorhabditis elegans* Maupas and nematodes living in natural andosol in agriculture soil. After treatment, they analysed the survival rate of nematodes using the Baermann funnel extraction technique and found that the average lethal treatment time with the surface barrier discharge ozone generator was 32s, while the ozone concentration was 32 g/m3. Tian et al. [45] tested the anti-ageing properties of cold plasma treatment in *C. elegans* and improved anti-ageing performance, including prolonged lifespan, activated movement behaviour and improved resistance to stress. To date, there are no records of tests of the effect of PAW on EPNs. Therefore, in our study, we tested the effect of PAW on selected EPNs for the first time. This is done to understand the interaction of EPNs, that are used as biological control agents; with PAW and whether the both can be recommended as an integrated pest management strategy against different soil-dwelling plant insect pests and plant diseases.

## 2 Materials and methods

### 2.1 Procurement of EPN

The EPN species were commercially available products of Biocont (CZ). *Heterorhabditis bacteriophora*, *Steinernema feltiae* and *Steinernema carpocapsae*. The products were stored in the refrigerator at 5 °C as per the instructions provided by the company (between 4 and 8 °C), until they were used for further experiment.

### 2.2 Preparation of PAW

**Fig. 1:**
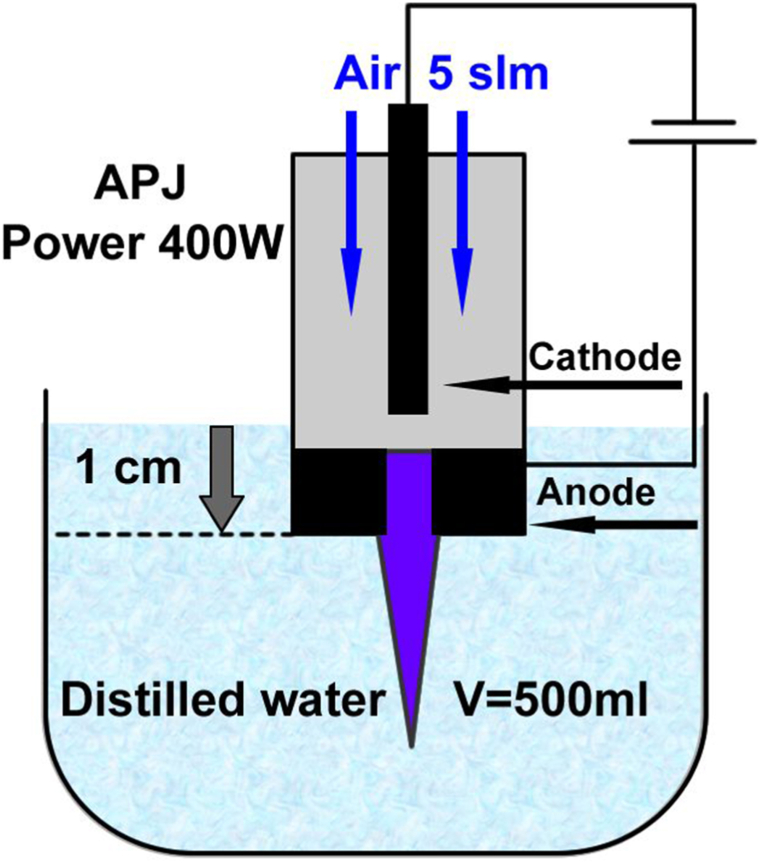
Schematic representation of the experimental set-up for APJ treatment of water.

PAW was generated using a DC air plasma jet (APJ) system as reported in Refs. [40,46] with minor modifications. Plasma was generated between a rod cathode and a cylindrical tube anode, separated by a neutrode. The cathode, made of copper with a hafnium insert, was connected to a regulated high-voltage power supply through a ballast resistor. The discharge was powered by a modulated DC voltage of 200 V and a current of 2 A. PAW was produced by immersing the plasma jet into 500 ml of distilled water in a Simax Lab Bottle GL80 to a depth of 1 cm, as shown in Fig. 1. The plasma jet was set to a power of 400 W with an air flow rate of 5 slm (standard liter per minute). The plasma treatment times in distilled water were 1s, 3s, 5s, 10s, 20s, 60s, and 90s. After the treatment, the PAW samples were taken to the Department of Environmental Ecology and Landscape Management at the Faculty of Natural Sciences for further experimentation. The nematodes were treated after 20 min post-PAW preparation.

### 2.3 Characteristics of PAW

The RONS namely peroxides (H_2_O_2_), nitrites (NO_2_^−^) and nitrates (NO_3_^−^) concentrations produced in PAW were characterized and measured using the exact protocol followed by Medvecká et al. [46].

### 2.4 *In vitro* test with EPNs

The procedure is a revised modification of Petrikovszki et al. [47]. The test was performed in the flat-bottomed 96-well plate under laboratory conditions. In each well, 10 alive infective juveniles in 40 μl distilled water of each species were carefully selected and placed into the well using a micropipette. Their viability was checked based on the movements before introducing in the well. One hundred and 10 μL of different PAW prepared at different treatment times (1s, 3s, 5s, 10s, 20s, 60s and 90s) were added to the well using micropipette. Distilled water was used as a negative control (0s). The microplates were covered with lid to reduce the interaction of air with the PAW and to avoid drying out of wells and were incubated in a thermostat in dark conditions at 20 °C ± 1 °C. Each treatment was replicated 8 times. The entire experiment was repeated twice. The plates were checked after an exposure period of 24 h under a transmission stereomicroscope. Juvenile mortality, if any, was recorded for each treatment after 24 h. The juveniles were considered dead when their entire body were motionless positioned in a straight line. A 20 % of maximum mortality was considered as a validity criterion for the tests in the control treatment [48].

### 2.5 Statistical analysis

Each nematode's data were processed and square root arcsine-transformed in an excel spreadsheet. One-way ANOVA post-hoc Tukey test was performed on the data based on whether the normality (Shapiro–Wilk test) was fulfilled in R software [62].

## 3 Results

### 3.1 Characteristics of PAW

**Fig. 2:**
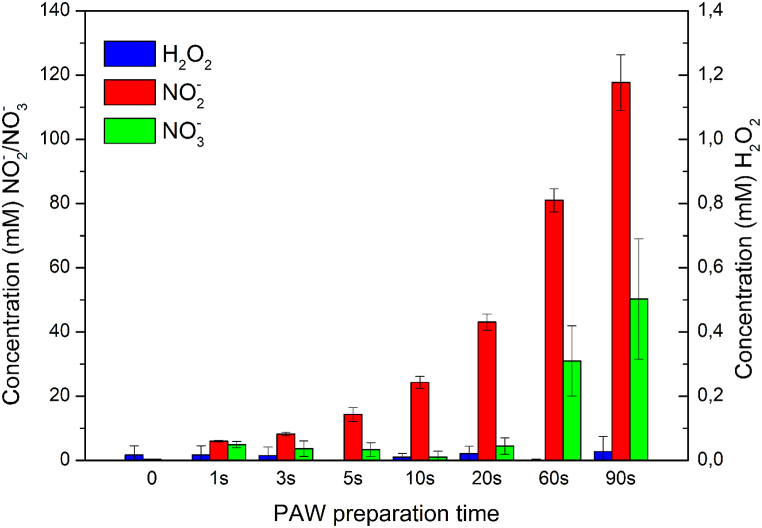
Concentration (mM) of reactive species measured in different plasma treatment time for producing PAW.

The concentrations (mM) of hydrogen peroxide, nitrates and nitrites produced in PAW prepared at different treatment times are shown in Fig. 2. The concentration of hydrogen peroxide was below the detection limit and was found to be in the range of 0.0167–0.027 mM and had almost the same value as the control, NO_3_^−^ in the range of 0.054–50.24 mM and NO_2_^−^ in the range of 0.33–117.7 mM.

### 3.2 *In vitro* test with EPNs

**Fig. 3:**
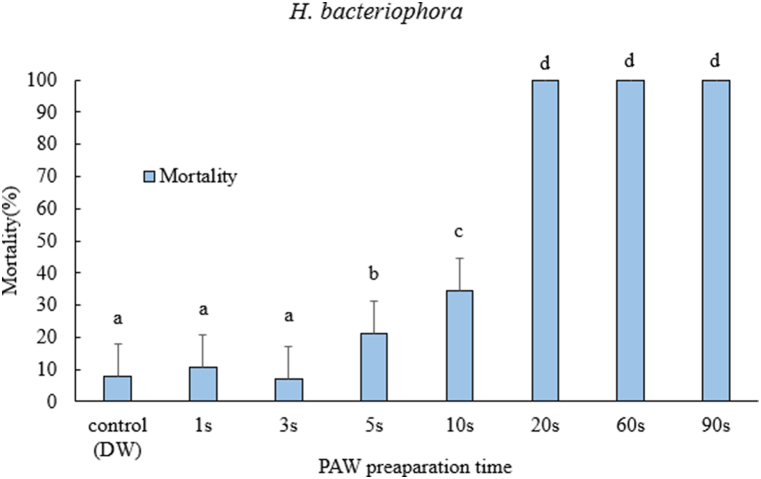
Mortality after 24-h exposure time of *Heterorhabditis bacteriophora* juveniles after treated with PAW prepared at different treatment time. One-way ANOVA post-hoc Tukey test was performed on the data (F= 260.2, df=7, p<0.05). The error bars indicate standard error of mean mortality. Different letters on top of the error bars indicate statistically significant difference at 95% confidence level.

In the case of *H. bacteriophora*, the mortality of the juveniles increased with increasing PAW preparation times (F = 260.2, df = 7, p < 0.05). The significance of mortality was observed from PAW prepared from 5 to 90s (Fig. 3, Table 1). PAW treated with 20, 60 and 90s exhibited 100 % mortality.

**Table 1:** ANOVA results of the effect of PAW prepared at different treatment times on the three nematode species, with p value significantly different at 95 % confidence interval. (Df = Degrees of freedom. Sq = square).

Species	Df	Sum Sq	Mean Sq	F value	p value
*H. bacteriophora*	7	73.33	10.47	260.2	<0.05
*S. carpocapsae*	7	2.694	0.3849	13.87	<0.05
*S. feltiae*	7	68.32	9.76	277.7	<0.05

**Fig. 4:**
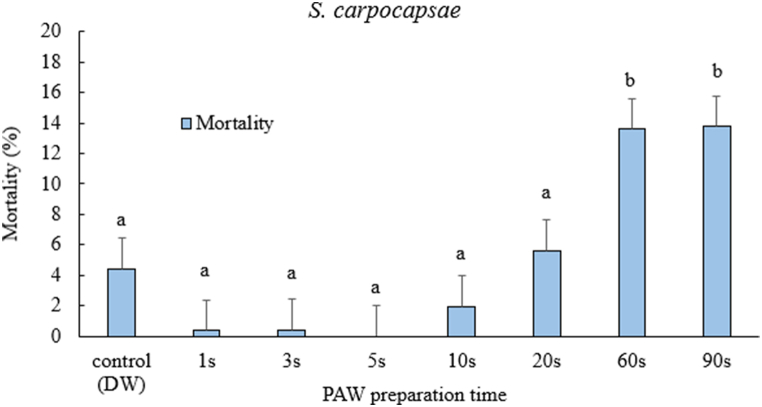
Mortality after 24-h exposure time of *Steinernema carpocapsae* juveniles after treated with PAW prepared with different time. One-way ANOVA post-hoc Tukey test was performed on the data (F= 13.87, df= 7, p<0.05). The error bars indicate standard error of mean mortality. Different letters on top of the error bars indicate statistically significant difference at 95% confidence level.

The mortality of the increased of *S. carpocapsae* juveniles significantly with the increase in the time to prepare PAW (Fig. 4, Table 1), the highest mortality in the case of PAW prepared for 60s and 90s was less than 20 %, which was the criterion selected to see any significant difference (F = 13.87, df = 7, p < 0.05). It appears that the juveniles of *S. carpocapsae* were not affected and were able to withstand the longest PAW preparation time.

**Fig. 5:**
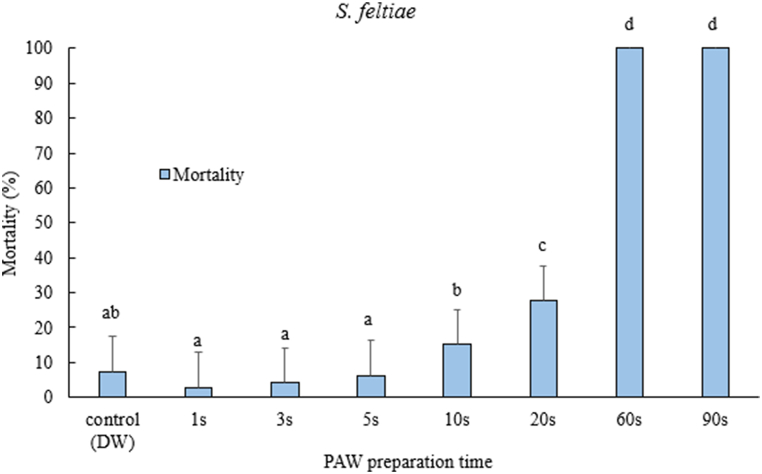
Mortality after 24-h exposure time of *Steinernema feltiae* juveniles after treated with PAW prepared with different time. One-way ANOVA post-hoc Tukey test was performed on the data (F= 13.87, df= 7, p<0.05). The error bars indicate standard error of mean mortality. Different letters on top of the error bars indicate statistically significant difference at 95% confidence level.

In the case of *S. feltiae*, a similar trend was observed in the mortality of juveniles as *H. bacteriophora* (Fig. 5, Table 1). However, *S. feltiae* showed significantly higher mortality only from PAW treated at 10s and above (F = 277.7, df = 7, p < 0.05). The 60s and 90s of PAW treatment showed 100 % mortality of *S. feltiae* juveniles.

## 4 Discussion

It is evident that the concentration of hydrogen peroxide was negligible, while the content of nitrites and nitrates increased with the increasing treatment time. The lower concentration of hydrogen peroxide in the APJ system for producing PAW could be due to the bubbles produced with high area of contact gas phase with water leading to effective dissolving in water [46]. Xiang et al. [49] suggested that the low concentration of hydrogen peroxide could be due to the interaction between hydrogen peroxide and nitrite ions under acidic conditions through the following reaction (1):

(1) NO_2_^−^ + H_2_O_2_ + H^+^ → ONOOH + H_2_O

Additionally, the shorter treatment time could be one of the possibilities for the lower concentration of hydrogen peroxide in PAW. Similar low concentrations of H_2_O_2_ were observed in PAW prepared after 30 min of plasma treatment of 800 ml of distilled water to decontaminate *E. coli* from the surface of soybean seeds [40].

In contrast to hydrogen peroxide, nitrogen oxides were found to be significantly higher (Fig. 2). This is because nitrogen oxides interact with water to form RNS. Air, as gas inlet, in the APJ system produces more RNS compared to hydrogen peroxide [46,50,51], which can also be seen in our results (Fig. 2). In the RNS produced, we found that the NO_2_^−^ concentration was higher compared to NO_3_^−^ in PAW (Fig. 2). The concentrations of both NO_2_^−^ and NO_3_^−^ increased with increased treatment time. Lin et al. [52] reported similar results, as they suggested that the volume of plasma-treated water may play an important role in the generation of the concentration of RNS and ROS. In our study, we treated 500 ml of distilled water with an APJ discharge system, fed by an air flow of 5 slm, and found similar results. Our results were also in parallel with Medvecká et al. [46], where they treated 200 ml of distilled water with the 5 slm of injected air with APJ system and found a significantly increased concentration of NO_2_^−^ and NO_3_^−^ compared to hydrogen peroxide. The different interactions of molecules and other particles were summarized while producing PAW by Ref. [53]. Another possible reason for high nitrate content could be that in the liquid phase, nitrates are formed at the expense of hydrogen peroxide, as reported by Machala et al. [54] through the following reaction (2):

(2) 2NO_2_ + H_2_O_2_ → 2NO_3_^−^ + 2H^+^

There was species-specific variation in the response of nematodes tested with PAW prepared at different treatment times. Our results from the interaction of the nematode with PAW demonstrate that there was variability in response to the RONS produced in PAW. The concentrations of both nitrites and nitrates proved to be strong determinants in the case of juvenile mortality of *H. bacteriophora* and *S. feltiae*. Previous studies of testing nonthermal plasma against nematodes included the effect of ozone generated from surface barrier discharge [55]. They tested the effect of ozone on *C. elegans*. They found that *C. elegans* was directly proportional to increase in ozone concentration. However, in our study, we did not measure ozone concentrations, but a similar trend was observed with NO_2_^−^ and NO_3_^−^ concentrations in PAW. In our case, the system for generating plasma and reactive species was different from the study by Mitsugi et al. [55].

These results could also be extrapolated to studies in which EPNs are exposed to different nitrogen-containing inorganic fertilizers. Bednarek and Gaugler [56] tested the compatibility of soil amendments with EPN. Their study demonstrated that *H. bacteriophora* was more sensitive to combined nitrogen (N), phosphorus (P) and potassium (K) amendments. In our results, we found that *H. bacteriophora* was the most sensitive of the three nematode species to PAW prepared from the 5s to the 90s. Similar results were also reported by Şahin et al. [57] when exposed to different inorganic fertilizers. This could be attributed to the genetics of the nematode, as well as to the smaller size of *H. bacteriophora* compared to *S. carpocapsae* and *S. feltiae* (Bednarek and Gaugler [56]. Perhaps, the size of *S. carpocapsae* could also be one of the reasons for less sensitivity to increased concentrations of NO_2_^−^ and NO_3_^−^ from PAW prepared at different treatment times. Another possible reason for the low mortality of *S. carpocapsae* could be its ability to different stressed environments [58].

This is the first report of testing the effect of PAW on EPNs. It is empirical to understand the compatibility of PAW with different nontarget organisms. Plasma activated water and EPNs have been used separately against common plant insect pests such as *T. urticae* by Savi et al. [42] and Abou El Atta et al. [59], respectively, against *P. citri* by Ten Bosch et al. [41] and van Niekerk and Malan [60], respectively, and against *F. occidentalis* [43,61], respectively. In this article, we propose a new integrated pest management strategy for testing PAW and EPN against different insect pests. However, we strongly recommend performing synergistic trials between different PAW preparations and EPNs before testing them against a common plant insect pest in future studies.

## CRediT authorship contribution statement

**Pratik Doshi:** Writing – original draft, Visualization, Validation, Software, Methodology, Investigation, Funding acquisition, Formal analysis, Data curation, Conceptualization. **Matej Klas:** Writing – review & editing, Methodology, Investigation. **Stanislav Kyzek:** Writing – review & editing, Investigation. **Anna Zahoranová:** Writing – review & editing, Supervision, Funding acquisition. **Božena Šerá:** Writing – review & editing, Supervision, Conceptualization.

## Ethical statement

Not applicable.

## Data availability statement

All the relevant data generated during this study are included within the article's figures and tables.

## Funding

This work was supported by Rector of 10.13039/100007594Comenius University Bratislava project titled “Synergy Effect of Biological Control and Non-Thermal Plasma Treatment against Fusarium Head Blight in Winter Wheat”. This work was supported by the 10.13039/501100005357Slovak Research and Development Agency, Slovakia under project no. APVV-23-0522 and APVV-21-0147.

## Declaration of competing interest

The authors declare that they have no known competing financial interests or personal relationships that could have appeared to influence the work reported in this paper.
